# The role of self-esteem, optimism, deliberative thinking and self-control in shaping the financial behavior and financial well-being of young adults

**DOI:** 10.1371/journal.pone.0256649

**Published:** 2021-09-07

**Authors:** Fatima Hashmi, Hira Aftab, José Moleiro Martins, Mário Nuno Mata, Hamza Ahmad Qureshi, António Abreu, Pedro Neves Mata

**Affiliations:** 1 Institute of Business and Information Technology, University of the Punjab, Lahore, Pakistan; 2 Lisbon Polytechnic Institute (IPL), Lisbon, Portugal; 3 (ISCTE-IUL), Business Research Unit (BRU-IUL), Lisbon, Portugal; 4 Lisbon Accounting and Business School Lisbon Polytechnic Institute, Lisbon, Portugal; 5 Polytechnic Institute of Santarém, School of Management and Technology (ESGTS-IPS), Santarém, Portugal; 6 Instituto Superior de Engenharia de Lisboa (ISEL), Instituto Politécnico de Lisboa, Lisboa, Portugal; 7 CTS Uninova, Faculdade de Ciências e Tecnologia, Universidade Nova de Lisboa, Lisboa, Portugal; 8 ESCS—Escola Superior de Comunicação Social, Lisbon Polytechnic Institute, Lisbon, Portugal; 9 ISTA—University Institute of Lisbon (ISCTE-IUL), Lisbon, Portugal; International Centre for Integrated Mountain Development (ICIMOD), Kathmandu, Nepal, NEPAL

## Abstract

The sustainable financial behavior and financial well-being have been a key concern among the developing societies; thereby encompassing the various psychological factors which play a role in influencing individual’s positive financial behavior and financial well-being, this study is conducted. Research focusing on the psychological aspect of human financial behavior and well-being is scarce, focusing more on the cognitive side such as financial literacy and numeracy. The aim of this research study is to find the role played by the non-cognitive factors such as self-esteem, self-control, optimism and deliberative thinking, in forming the financial behavior and financial well-being of the young adults. A sample of 429 university students from public and private sector was collected via an online and field survey using purposive sampling technique. The survey contained measures for demographics, self-esteem, optimism, deliberative thinking, self-control, general financial behavior and financial well-being. SPSS and PLS-SEM tools were used for the exploration of the relationships among dependent and independent variables. The results of PLS path analysis demonstrate that among the non-cognitive factors, self-control and deliberative thinking show a significant association with both financial behavior, and financial security. Self-esteem plays no significant role in forming the financial behavior of the young adults when all the variables are taken together but it exhibits a significant association with financial well-being (financial security and financial anxiety). Optimism on the other hand exhibits no significant association with both financial behavior and financial well-being (financial security and financial anxiety). The results of this study complement the previous studies and also put forth new outcomes. This research is unique as it is the first of its kind conducted in a consumption-oriented economy like Pakistan. In addition to the previous studies which have often established the link of self-esteem with general well-being, this study goes further by analyzing the association between self-esteem and financial well-being and by the identification of the role played by non-cognitive factors like self-esteem, optimism, deliberative thinking and self-control together on the financial behavior and financial well-being of the individuals using PLS-SEM approach.

## 1. Introduction

The consumption expenditure pattern of the Pakistani households has increased by 14%, whereby the average per capita expenditure has increased from Rs.5166 ($32.24) to Rs.5959 ($37.19) (2018–19), since the last survey conducted during the annual period 2015–16 [1]. According to one study, about one-fifth of the Pakistani households on average are indebted and about 40% of the over-indebted households spend more than 100% of their income on the repayment of liabilities [2].

Strömbäck and collegues [3] in their study state that people make poor financial decisions. People consume or spend more than what they earn, they fall behind in paying their bills or liabilities on time or they save very little for the future needs such as for retirement. Individuals sometimes spend on things which they would regret later, or they fail to save for achieving some long-term objective (like a vehicle, house, higher education, etc.). However, we as individuals vary in exhibiting this behavior, a few of us may tend to make somewhat bad financial decisions and be more or less susceptible to the anxiety caused by it. To understand this behavioral heterogeneity among individuals and to lessen this development of increasing consumption expenditure patterns and over indebtedness, it is important to identify the factors behind these poor financial behaviors and ensuring well-being of the individuals.

One of the reasons associated with this poor financial behavior is the lack of financial literacy. Financial literacy is not only the knowledge of financial concepts but also the ability of the individuals to use this knowledge for making effective financial decisions [4]. This intuitive connection may provide one of the ground bases as for why most of the previous researches have focused on studying financial literacy, suggesting the lack of it to be the cause of poor financial behavior [5, 6], but there are other aspects to the relation. Confirming to the outcomes of a meta-analysis conducted by Fernandes and his colleagues [7], financial education used as a mediator demonstrated only 0.1% variance in financial behavior, implying it to be an insufficient factor in improving the financial behavior. Their study further suggested that a substantial reduction is observed in the relation among financial literacy and financial behavior when the psychological factors are under control. Same results were obtained from the study of Bapat [8] showing that no direct relation exists between financial knowledge and financial behavior. For the purpose of understanding the financial decision making ability of the individuals, the study of the underlying psychological characteristics is important, influencing both financial behavior and financial well-being [3]. According to Tang and Baker [9], financial behavior is driven by financial knowledge that is vital yet inadequate in explaining the financial behavior alone; an important role is played by the definite evaluation of an individual in this process.

Literature suggests that psychological factors play an important role in the financial behavior and well-being of the individuals. Among them is self-esteem which is defined as one’s overall self-perception and has been found to be associated with subjective well-being [10] and financial behavior both explicitly or implicitly via subjective financial knowledge [9]. Though self-esteem is one of the most popular human traits that has been widely studied in vast applied psychological and personality studies yet very little work is done in respect of its association with financial behavior [9] and financial well-being. Self-control is another important personality trait which has received much attention. People with high self-control save more, exhibit responsible financial behavior, experience less anxiety regarding one’s financial situation. Lack of self-control leads towards poor financial behavior (compulsive buying) which is strongly linked to low levels of self-esteem and optimism [11]. In addition to self-esteem and self-control two other psychological factors are of interest, optimism and deliberative thinking. Dispositional optimism—where an individual has a positive general outlook for the future, has been demonstrated by vast research as advantageous for psychological and physical well-being [12]. Optimistic individuals work harder, save more, feel more secure and less anxious about their financial situations while extreme optimism has been identified to have negative effects on the financial behavior [12, 13]. According to Dual Process Theory, individuals usually take decisions based on intuition (system 1) but sometimes intuition may be overridden by deliberative thinking (system 2). The choice of either system not only has an impact on the behavior (action) but also on the well-being [14]. Intuition should be used when an individual is certain about a situation and has expertise [15]. Based upon the literature we have limited the scope of this study to focus only on the deliberative thinking (system 2) of the individuals in their financial decision making and since in this study we are also focusing on self-control which is a system 2 driven trait [14].

Scant research is available focusing on features like self-control, self-esteem, deliberative thinking and optimism, influencing an individual’s financial behavior and financial well-being. In this study, unlike most of the previous studies, we aim to look at a more general financial behavior such as payment of liabilities or bills on time, comparison shopping, saving for some long-term goals, investing and insuring. This approach is more beneficial as it includes the actions and decisions individuals make in their daily lives. As the young adults start gaining independence in their decisions in both general and financial matters, they do not aim at saving for their retirement, neither they have money for investment (often), and at young age they do not have any liabilities on them. Therefore, in order to obtain a complete overview, the aspects that affect an individual’s financial behavior are explored as a primary goal of the research.

In South Asian countries (like Pakistan) as long as an individual is a student, his/her parents bear all the expenses (in majority of the cases). There is a family system in place where children especially females, regardless of reaching maturity (18+) still depend upon their parents or families. Though this trend is now changing as a large number of young adults are engaging in some sort of money-making businesses such as part time jobs or free-lancing. In-short the general set-up of the young adults in a culture like Pakistan’s is quite different from those of the western cultures where such studies exploring the relationships of psychological traits, behaviors and well-being have been conducted. Thus, the second aim of the study is exploring the factors effecting the financial well-being of the individuals.

In view of these and many other differences there is a possibility of variations in the outcome of this study and this is one of the reasons for conducting this research. Since the relation among financial well-being and self-esteem has not been investigated in particular, this study fills that gap with the inspection of the influence of these non-cognitive factors in shaping the financial behavior and financial well-being of the individuals studying in private and public sector universities of Pakistan.

Since, this research was conducted during a global pandemic, the average behaviors and the well-being of the individuals are expected to be affected by this havoc, affecting individuals both physically and mentally all around the world thus, influencing their behaviors and well-being.

The investigation of the various psychological traits relationship with financial behavior and financial well-being of the individuals is vital to highlight their importance; as subsequently necessary steps for focusing on the development of the non-cognitive skills of the young adults could be taken along with their basic financial literacy. This would support the individuals in making better financial decisions in life that will ultimately lead to a better well-being.

### 1.1 Non-cognitive factors, financial behavior and financial well-being

The focus of the previous researches has been mostly on the cognitive factors such as financial literacy [5] and numeracy [16] in shaping the financial behavior. Cognitive abilities include intellectual efforts like reasoning, thinking and remembering, etc. [17]. According to Parise and Peijnenburg [18], the measures most often used for measuring cognitive abilities are IQ and numeracy tests. On the other hand, non-cognitive is a term having multiple placements which includes traits, skills, abilities, attributes and outcomes. A countless number of other such specific skills are also recognized as non-cognitive. Among the frequently used are anxiety, attitude, self-efficacy, confidence and curiosity [19].

The present study focuses on the role of self-esteem, self-control, deliberativeness and optimism in forming individuals’ financial behavior and financial well-being. Any behavior of human beings related to money management can be defined as their financial behavior. Financial behavior commonly refers to cash, credit and saving behaviors [20]. Financial behavior encompasses four broad areas namely saving, borrowing, spending and investing. Individuals differ in their financial behaviors subject to different aspects such as identity, financial literacy, peer groups, family background, personal characteristics, income, performance, psychological factors, etc. [21–23]. In this study we aim to evaluate the general financial behavior of the individuals.

The possibility of people’s well-being is one of the biggest reasons behind the stimulation of better financial decisions. Financial well-being defined as “The extent to which someone is able to meet all their current commitments and needs comfortably, and has the financial resilience to maintain this in the future”, is considered by the people to be mainly an objective measure ([24], p.45). The financial domain of subjective well-being is one of the least understood. Firstly, given prior reliance on the objective measures such as income, little literature is available focusing on how an individual’s ability to control impulses such as that of anxiety effects one’s financial situation. Along with focusing on the objectivity of financial well-being, its equally important aspect is how people subjectively feel about their financial situations. An individual’s overall subjective well-being is greatly influenced by financial well-being [25]. Depending upon the individual characteristics even the individuals having the same financial statuses may differ in their respective levels of subjective financial well-being [26]. Considering the financial well-being of an individual, the subjective measures provide a greater explanation about it whereas more tangible and comprehensible interpretation is provided by the objective measures [27]. Secondly, the target population of the study i.e., the young adults often are not holding any jobs while they are still pursuing their education. They don’t have any source of income or own any assets except for that of their parents in most of the cases. Thus, in this study we have focused on subjective financial well-being. After the evaluation of anxiety and security related to finance, the computation of the financial well-being can be done through it, as demonstrated in the study of Stromback and his colleagues [3]. Ultimately, the advancement of well-being is one of the purposes of enhancing decision making and behavior.

Various studies have been conducted on young adults comprising of college/university students to know about their financial behavior and financial well-being. Financial capability does not only depend upon financial literacy or knowledge, but it can also be developed by non-formal financial socialization elements like peer groups or parents. Financial education, work, self-actualizing values, and parents all play a role in forming the financial attitudes and behaviors of the young adults but the role played by the parents in communicating the finances is found to be more substantial than both work, and education combined. Satisfaction of young adults with their financial situation leads to satisfaction in life in general which in turn enables them to succeed academically and be healthy both psychologically and physically [28, 29]. Salignac and colleagues in their study found that financial well-being does not only refers to the financial circumstances of the young adults but it also depends upon their interaction with the environment–social, community, life-course stages and both predicted or unpredicted financial shocks [30]. Lanz, Sorgente and Danes conducted a study to learn about the impact of implicit family financial socialization on the emerging adult’s financial well-being. In the study an indirect effect of the quality of family communication was found on the subjective financial well-being of the young adults depending upon the degree to which they model their financial behaviors as that of their parents. This shows that not only the communication about finances is important but also the quality of it, for the financial development of the young adults [31]. Similarly, in another study the authors highlight the importance of quality financial parent-child communication as it leads towards positive subjective well-being (financial, personal, and psychological) and development of sound financial coping behaviors of the young adults. Acquisition of sound financial behaviors may contribute towards the financial competency and self-sufficiency of the youngsters in adulthood [32]. According to one study, it was found that student’s high financial well-being is associated with low levels of financial anxiety regardless of the type of the debt held by them [33]. In another research highlighting the financial instability in emerging adulthood, it was found that the subjective financial well-being of the college students is negatively associated with student debts starting from the 1^st^ year of the college and proceeding even in the early years after its completion [34]. Paola Iannello and colleagues in their study on the subjective financial well-being of young adults found that the individual differences in tolerance to ambiguity and uncertainty about the current and future financial situations play a moderating role in the relationship between the subjective financial well-being and psychological well-being of the young adults. While the subjective financial well-being of the emerging adults plays an invariant role in the subjective well-being of the individuals, irrespective of their individual differences. The more an individual is satisfied with his/her financial condition leads towards overall perceived life satisfaction [26]. Self-esteem plays a major role in enabling the individuals to make sound financial decisions. The more a young adult believes in his/her ability to manage the finances the better his financial behavior is. Serido, Shim and Tang (2013) [35] in their study showed that financial self-efficacy influences young adults’ financial behaviors and as they practice these financial behaviors it rises their levels of financial well-being and overall well-being. Thus, these studies highlight the importance of the individual characteristics for developing sound financial behaviors and financial well-being in young adults along with their financial education.

#### 1.1.1 Self-esteem

One of the popular psychological subject since decades is self-esteem [36–39]. Self-esteem is literally described as how much worth individuals put on themselves. It is a component used to analyze self-knowledge [40]. Baumeister and colleagues [41] in their study (monograph) summarized the benefits of high self-esteem under two themes: high self-esteem makes one feel good and confident and it fosters initiative. People with high self-esteem unlike their counterparts exhibiting low levels of self-esteem, don’t give up easily in time of stress and are more prone to both prosocial and antisocial behaviors. However, it was found that self-esteem does not lead to good school performance and increasing the self-esteem may prove to be counterproductive.

An imperative predictor of subjective well-being as shown by the previous studies is self-esteem. Financial self-efficacy positively impacts savings, insurance, investment and budget dimensions of financial behavior which in turn positively impact financial well-being [42]. In relation to financial behavior including risky investments, savings and management of credit, self-esteem was found to have both direct and indirect (via subjective financial knowledge) positive relationship [9]. Similarly, Ramalho and Forte [43] in their study found that high levels of self-esteem lead to better financial behavior of the individuals and also that self-esteem mediates the relationship between financial knowledge and financial behavior. Self-confidence in one’s financial management skills plays a vital role in the financial inclusion i.e. the access to and usage of financial services and products [44]. Neymotin [45] using a selected sample of individuals, explored the relationship between individual’s self-esteem and their propensity to involve in different financial planning measures including planning retirement, record keeping of budget and record keeping of the charges of credit card. The results demonstrated that self-esteem has a vital role in financial planning decisions. Similarly, self-efficacy was found to successfully mediate the relationship between credit card literacy of the college students and their financial well-being [46].

#### 1.1.2 Optimism

According to Carver, Scheier and Segerstrom [47], individuals expecting good in life are optimists; whereas people who expect bad circumstances in life are pessimists. Puri and Robinson [12] exploring the association between optimism and individual economic decision making, found that optimistic individuals save more, work harder, participate in the equity market, are more likely to buy individual stocks and retire later. Optimism in moderation leads to more prudent financial decisions and greater self-control unlike extreme optimism which correlates with unwise financial habits and behavior. Optimists tend to be less anxious and more secure in their financial matters and situations, respectively and observe improved financial behavior [3]. Similarly, optimism was found to be negatively associated with financial anxiety while positively with financial security showing that optimists feel more financially secured and worry less about their financial positions [13] but, unlike the previous studies which highlight a positive association between individual’s optimism and their financial behavior, the results of Hirvonen [13] and Ianole-Calin and colleagues [48] research studies, showed that being optimistic about the future did not help one prepare for it and thus no relationship was observed between optimism and financial behavior.

#### 1.1.3 Deliberative thinking

The ability of humans to make decisions has been described utilizing two kinds of interacting systems, System 1 (intuition) is speedy, automated, and straightforward, whereas System 2 (deliberative thinking) is steady, supervised, and takes effort. System 1 rapidly offers answers, and System 2 observes System 1, aids in resolving those problems for which the solution is not given at once, also checks and re-evaluates for any mistakes in System 1 [49, 50].

Financial traders use fewer heuristics in decision-making and are more prone to deliberative thinking than intuition or gut feeling as compared to non-financial traders [51]. Moxley, Ericsson, Charness and Krampe [52] in their study on strategic decision making of chess experts found that deliberative thinking supports intuitive decision making, enabling the individuals to make the best moves. From their study they inferred that both experts and non-experts benefit with added deliberative thinking whether they are to make easy or difficult decisions in any domain of expertise, such as in financial decision making. Investors make use of different non-conventional decision-making heuristics, both intuitive (fast thinking) and deliberative thinking (slow thinking) to cope with the bounded rationality of the world. This helps to achieve optimal results unlike the neoclassical decision making. However, fast thinking sometimes results in suboptimal outcomes, depending upon the opportunities available to the investors and the decision making environments [53]. Furthermore, sound financial behavior and perceived financial security was observed in individuals scoring high on deliberative thinking while it showed no influence on financial anxiety [3]. Similarly, a positive effect of deliberative thinking was observed on the financial behavior (saving) and financial security while no effect was observed in case of financial anxiety, in a study conducted by Ianole-Calin and his colleagues [48].

#### 1.1.4 Self-control

Self-control is outlined as a person’s ability to reform a primary response to something or convey an appropriate response. It allows people to avoid giving an immediate reaction, thereby keeping an adaptive behavior. This means that through self-control, we can concentrate better and not give way to unwanted thoughts, we can hold on to our instant temptation of eating a piece of chocolate cake thereby delaying instant gratification, regulating our emotions when confronted with a situation, or improve at something with consistent practice [54]. Mischel and his colleagues [40] conducted three different self-control experiments (marshmallow and pretzel tests) on pre-school children. Majority of the children exhibited low self-control by not resisting the temptation. Mischel followed-up this sample of the pre-schoolers and found how the ability of the children who demonstrated self-control early in life correlated with their life outcomes as they grew old. Such children were reported to be more academically and socially competent, scoring high in SAT, they were expressive, skillful and confident. They were better able to cope up with problems and think ahead of time.

In relation with financial behavior, numerous literature is available highlighting the importance of self-control. Strömbäck and collegues [3] in their study on a large scale Swedish population (n = 2063) found that self-control influences both the financial behavior and financial well-being of the individuals. It was found that people with high self-control saved regularly, instill good financial behavior, exhibited less anxiousness and felt more secure in financial situations. Similarly, Hirvonen [13] conducted an online survey in Finland to study how the financial behavior and well-being of the university students were effected by their self-control and optimism. The results demonstrated that high self-control enabled the individuals to save more and be prepared for the future while low self-control was linked with poor financial behaviors. Furthermore, individuals having high self-control exhibited low debt and less compulsive buying problems than individuals lower in self-control [11].

Consumer over-indebtedness is more strongly explained by the absence of self-control than the lack of financial literacy. Individuals having low self-control in financial realm are exposed to a variety of risks leading to over indebtedness such as credit withdrawals and credit shocks [55]. Subjective and objective self-controls are two separate entities. A positive association of financial behavior and financial well-being was identified with subjective self-control but not with the objective self-control which points out that the individual’s ability of resisting financial temptations is more imperative than cognitive in managing sound financial behavior and financial well-being [56].

Previous researches on personality traits has mostly been conducted in the developed countries where the educational level and the general well-being of individuals is better than those in the developing countries like Pakistan. In-short the general set-up of the young adults in a culture like Pakistan’s is quite different from those of the western cultures where such studies exploring the relationships of psychological traits and behaviors have been conducted. Moreover, in moving from one region to another, the cultural differences dominate which might influence the study. Through this study we aim to explore and analyze the role different psychological factors have in shaping the financial behavior and financial well-being of the young adults. This information could ideally provide some support in understanding and devising ways to improve the financial behavior and financial well-being of the individuals. Moreover the current research aims to make additions in the literature and complement the existing work, providing guidance for future research in this area.

**Fig 1:**
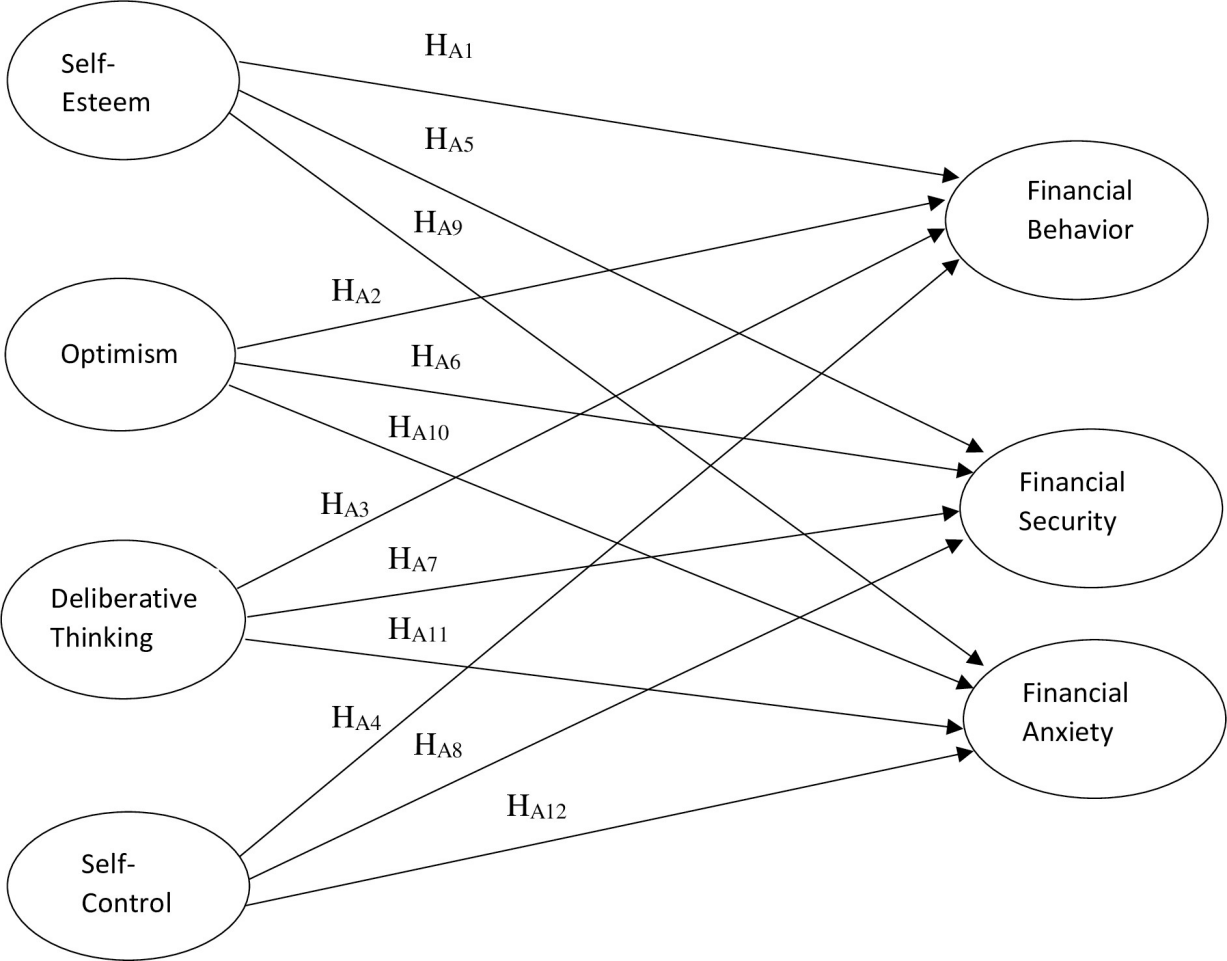
Conceptual framework of the study.

The conceptualized framework of the study based upon the literature is presented below in Fig 1.

### 1.2 Research objectives, research questions and hypothesis

This research study aims at exploring: “Do non-cognitive factors such as self-esteem, self-control, optimism and deliberative thinking play a role in shaping the financial behavior and financial well-being of the individuals?”

The aim of this study was divided into two research questions which were measured through the formulation of multiple hypotheses.

RQ_1_: Do non-cognitive factors contribute in forming the financial behavior of the young adults?
H_01_: There does not exists a significant relationship between financial behavior and self-esteem.
H_A1_: There exists a significant relationship between financial behavior and self-esteem.
H_02_: There does not exists a significant relationship between financial behavior and optimism.
H_A2_: There exists a significant relationship between financial behavior and optimism.
H_03_: There does not exists a significant relationship between financial behavior and deliberative thinking.
H_A3_: There exists a significant relationship between financial behavior and deliberative thinking.
H_04_: There does not exists a significant relationship between financial behavior and self-control.
H_A4_: There exists a significant relationship between financial behavior and self-control.
RQ_2_: Do non-cognitive factors contribute in forming the financial well-being of the young adults?
H_05_: There does not exists a significant relationship between financial security and self-esteem.
H_A5_: There exists a significant relationship between financial security and self-esteem.
H_06_: There does not exists a significant relationship between financial security and optimism.
H_A6_: There exists a significant relationship between financial security and optimism.
H_07_: There does not exists a significant relationship between financial security and deliberative thinking.
H_A7_: There exists a significant relationship between financial security and deliberative thinking.
H_08_: There does not exists a significant relationship between financial security and self-control.
H_A8_: There exists a significant relationship between financial security and self-control.
H_09_: There does not exists a significant relationship between financial anxiety and self-esteem.
H_A9_: There exists a significant relationship between financial anxiety and self-esteem.
H_010_: There does not exists a significant relationship between financial anxiety and optimism.
H_A10_: There exists a significant relationship between financial anxiety and optimism.
H_011_: There does not exists a significant relationship between financial anxiety and deliberative thinking.
H_A11_: There exists a significant relationship between financial anxiety and deliberative thinking.
H_012_: There does not exists a significant relationship between financial anxiety and self-control.
H_A12_: There exists a significant relationship between financial anxiety and self-control.

## 2. Data and methodology

### 2.1 Data

The target population for the study constitutes of university students ranging from age 18 and above. The data for the study was collected via two means: a larger portion of which constituted of online survey, in view of Covid-19, and when the situation was a little under control then by physically distributing the copies of survey papers among university students. The language of the survey was English. Participants were informed about the purpose of the research and ensured that their information will only be used for research purposes. A formal written consent was taken from the participants both online and on field survey highlighting that the information acquired will only be used for the research purpose. The ones who agreed proceeded with filing the forms.

### 2.2 Sampling technique and sample size

Purposive sampling was used to gather the data. The sample was determined purposively by following the criteria that the unit sample should be a student studying at a university and is 18 years or above in age at the time of filling the survey Through online survey 379 responses were collected while 50 responses were collected through physical survey. A total sample of 429 university students was gathered which constituted of 257 public and 172 private sector university students. A sample size should be such that it gives the full representation of the population and it should neither be too small nor too large but optimum. Unfortunately, no accurate measure is available for selecting an appropriate sample size as it depends upon various factors but most of the researchers are of the view that bigger the sample size the better the results are [57]. Data normality issues are often associated with small sample sizes; according to a study, sample sizes of 200 or more are less likely to face normality issues [58]. Many researches also support the rule of thumb that for each construct there must be at least 10 observations. Depending upon the size of the target population (students enrolled in the universities) and taking 95% confidence interval, the minimum sample required for the study came out to be 384 using a sampling formula [59]. Thus, our sample size meets all the above criteria for a representative sample.

In the current study the number of female responses obtained was greater than the male responses, this can be due to the reason that males and females differ in the way they inhabit cyberspace which may lead to differences in the way they undertake social exchange thus resulting in differences in the rates of the survey response [60]. It has been observed by many researchers that females mostly engage in online activities that involve communication and exchanging of information while males tend to engage in online activities that involve seeking information [61].

The relationships among the non-cognitive factors with financial behavior and financial well-being were investigated using SPSS and PLS-SEM tools. Demographic analysis of the respondents (Table 1), and Pearson Correlation (Table 2) were carried out using SPSS. For path analysis, PLS-SEM technique was used. For the assessment of measurement model, composite reliability is reported. For the assessment of the structural model, collinearity assessment (VIF values), path coefficient, coefficient of determination (R square or R^2^ Value) and predictive relevance (Q^2^) is reported.

**Table 1:** Demographic statistics of the respondents.

Variable	Category	Distribution	
		Frequency	Percentage
Gender	Male	110	25.6
	Female	319	74.4
Age	18–22	204	47.6
	23–26	202	47.1
	27–30	13	3.0
	31–34	4	.9
	Above 34	6	1.4
Education	BS 2-years	7	1.6
	BS 4-years (hons)	195	45.5
	Masters	40	9.3
	Mphil	77	17.9
	Ph.D.	2	.5
	MBBS/BDS/Other	108	25.2
Type of University	Public	257	59.9
	Private	172	40.1
Year of Study	1st year	74	17.2
	2nd year	88	20.5
	3rd year	38	8.9
	4rth year	92	21.4
	Other	137	31.9
Household income (Monthly)	Less than Rs.25,000	25	5.8
	Rs.25,000—Rs.50,000	73	17.0
	Rs.50,001 -Rs.100,000	141	32.9
	Rs.100,001-Rs.200,000	100	23.3
	More than Rs.200,000	90	21.0

Note: n = 429; income is given in Pakistani rupees (PKR); Ph.D: Doctor of Philosophy; MBBS: Bachelors of Medicine and Bachelor of Surgery; BDS: Bachelors of Dental Surgery.

**Table 2:** Two-tailed correlations among predictors and dependent variables.

		Financial Behavior	Financial Security	Financial Anxiety
Self-Esteem	Pearson Correlation	.171**	.345**	-.216**
Optimism	Pearson Correlation	.114*	.153**	-.235**
Deliberative Thinking	Pearson Correlation	.179**	.275**	-.020
Self-Control	Pearson Correlation	.216**	.265**	-.161**

Note: * p < 0.05 level;

** p < 0.01

The relational strength among the dependent and independent variables was determined through the execution of Pearson correlation. The results of correlation demonstrate that self-esteem, optimism and self-control have a significant negative association with financial anxiety which depicts that with the increase in the levels of these psychological traits, financial anxiety decreases. While deliberative thinking does not exhibit a significant association with financial anxiety. On the other hand, with the increase in the levels of these traits, young adults demonstrate good financial behavior and increased financial security. The results of Pearson correlation show a positive one-to-one (linear) relationship among the independent and dependent variables, which is in line with the previous studies e.g. [3, 9].

### 2.3 Measures

The questionnaire used for conducting this study constituted of seven segments, where the first segment included questions pertaining to demographics (Table 1), such as age, gender, and monthly household income, etc. The proceeding sections contained scales to measure the constructs included in the research study, the details of which are given below:

#### 2.3.1 Self-esteem

To measure the self-esteem of individuals, one of the most popular self-esteem scale developed by Rosenberg [36] was used. There are 10 items on the aforementioned scale and it uses a likert scale. The options on likert scale range from 1 to 5 where 1 depicts “Strongly Disagree” and 5 depicts “Strongly Agree”. 5 out of 10 options in the scale were reverse coded like “I wish I could have more respect for myself” and “At times I think I am no good at all” as they are negative statements.

#### 2.3.2 Optimism

LOT-R short for ‘Life Orientation Test-Revised’ scale designed by Scheier, Carver and Bridges [62] was adopted to measure optimism. The scale consists of 10 items and the division of this scale is as follows: three items for evaluating optimism; three items measure pessimism and the rest of the four items are used as fillers and were not scored [62] thus only 6 items out of 10 were used to measure optimism. The items measuring pessimism were reverse coded like “I hardly ever expect things to go my way”. The participants were asked to score each item using likert scale ranging from 1 to 5 where 1 for “Strongly Disagree” and 5 for “Strongly Agree” was used.

#### 2.3.3 Deliberative thinking

For measuring the extent of individuals’ deliberative thinking, a unified scale developed by Pachur and Spaar [63] was used. This is a 16-point scale utilized for the preference of deliberation. The participants scored likert scale items from 1 to 5, where 1 depicted “Strongly Disagree” and 5 depicted “Strongly Agree”.

#### 2.3.4 Self-control

A scale designed by Tangney, Baumeister and Boone [64] for measuring Self-Control in individuals was used (Brief Self-Control scale). There are 13 items in this scale and 9 of them were coded in reverse as they are negative statements. Likert scale (5-point scale) was used for evaluating Self-Control; where 1 depicted “Not at all like me” and 5 depicted “Very much like me”.

#### 2.3.5 Financial behavior

General financial behavior of young adults was accessed using FMBS short form of ‘Financial Management Behavior Scale’ developed by Dew and Xiao [65], which evaluates common behavior related to financial activities, for instance timely bill payment, comparison shopping and keeping record of expenses rather than just focusing on the saving behavior of the individuals. This scale originally included 15 items measuring the individual’s financial behavior over the previous 6 months, one statement was excluded in our study i.e. “Maintained or purchased an adequate health insurance policy” because in Pakistan health insurance is covered in the life insurance which is the last item of the scale. Two of the items were reverse coded in the scale as they exhibit negative behavior. The behavioral pattern of the participants regarding financial activities, was measured using likert scale with options from “Never” = 1 to “Always” = 5. “Not Applicable” option was included for statements especially from statements 5 to 7 (related to credit cards and loan) as in Pakistan here is a minimum trend of owning a credit card and students are unlikely to owe any debts. The “Not-Applicable” option was coded as ‘1’.

#### 2.3.6 Financial well-being

Netemeyer, Warmath, Fernandes and Lynch [66] designed a scale for evaluating the financial well-being of the individuals. Two separate but related constructs, Current Money Management Stress (Financial Anxiety) and Financial Security, were used for the evaluation of financial well-being. Each of the constructs constituted 5 items. The participants used likert scale for rating the items ranging from 1 to 5 where 1 depicted “Does not describe me at all” and 5 depicted “Describes me completely”.

### 2.4 Ethical statement

This research study encompasses the involvement of human participants, thus ethical approval was taken after its review by the IBIT Research Council, University of the Punjab, Lahore.

## 3. Analysis and results

**Fig 2:**
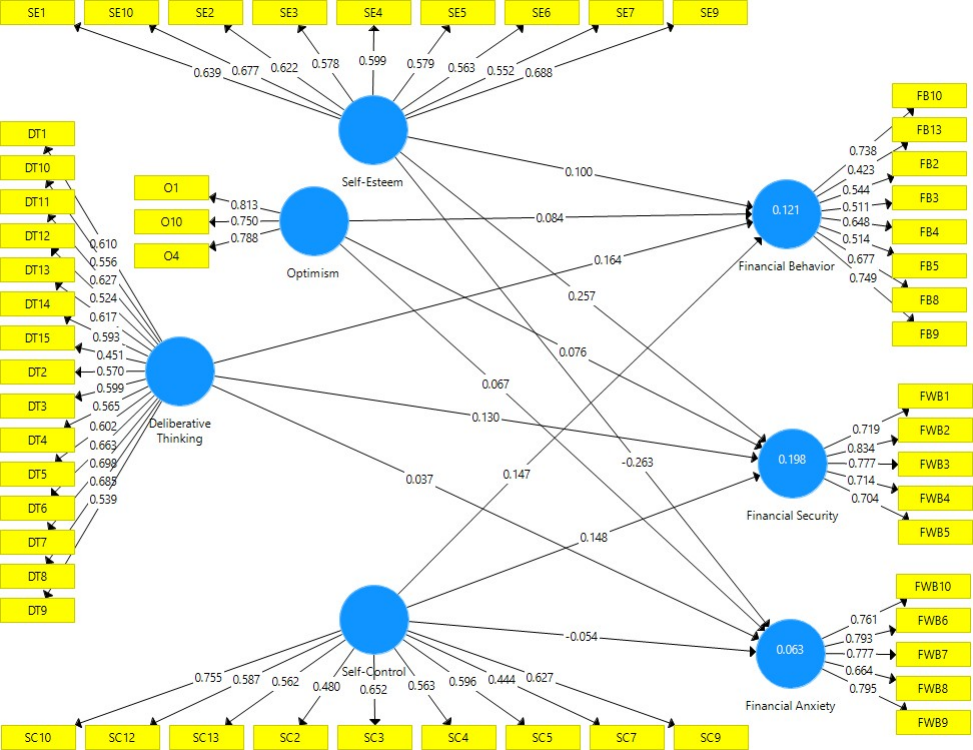
Estimated model.

When dealing with multiple variables, Structural Equation Modeling has advantages in path analysis and regression. For Path analysis, PLS-SEM technique is followed in the current study. The advantages of using PLS-SEM is that, it is more flexible with the sample sizes and is also less susceptible to the multivariate data assumptions violations such as normality of data [67]. The estimated model developed for the current study using PLS-SEM technique is given below Fig 2.

### 3.1 Assessment of reflective measurement model

First, items were scrutinized on the basis of sufficient outer loadings (Table 3), items having loadings λ < 0.4 were removed [68] and then the assessment of measurement model and structural model was done with the help of the remaining items. To measure the internal consistency of a scale, composite reliability is preferred over Cronbach’s alpha in PLS-SEM. It ranges from 0 to 1 where higher the value indicates higher the reliability. Table 3 shows that all the constructs have satisfactory reliability values (i.e. CR > 0.7).

**Table 3:** Measurement model.

Construct	Item-Code	Loadings (λ)	Composite Reliability (CR)
Self-Esteem			0.843
	SE1	0.639	
	SE2	0.622	
	SE3	0.578	
	SE4	0.599	
	SE5	0.579	
	SE6	0.563	
	SE7	0.552	
	SE9	0.688	
	SE10	0.677	
Optimism			0.827
	O1	0.813	
	O4	0.788	
	O10	0.750	
Deliberative Thinking			0.891
	DT1	0.610	
	DT2	0.570	
	DT3	0.599	
	DT4	0.565	
	DT5	0.602	
	DT6	0.663	
	DT7	0.698	
	DT8	0.685	
	DT9	0.539	
	DT10	0.556	
	DT11	0.627	
	DT12	0.524	
	DT13	0.617	
	DT14	0.593	
	DT15	0.451	
Self-Control			0.826
	SC2	0.480	
	SC3	0.652	
	SC4	0.563	
	SC5	0.596	
	SC7	0.444	
	SC9	0.627	
	SC10	0.755	
	SC12	0.587	
	SC13	0.562	
Financial Behavior			0.821
	FB2	0.544	
	FB3	0.511	
	FB4	0.648	
	FB5	0.514	
	FB8	0.677	
	FB9	0.749	
	FB10	0.738	
	FB13	0.423	
Financial Security			0.866
	FWB1	0.719	
	FWB2	0.834	
	FWB3	0.777	
	FWB4	0.714	
	FWB5	0.704	
Financial Anxiety			0.872
	FWB6	0.793	
	FWB7	0.777	
	FWB8	0.664	
	FWB9	0.795	
	FWB10	0.761	

### 3.2 Assessment of structural model

High correlations among variables leads to a collinearity problem. The collinearity among the variables was assessed through Variance inflated factor (VIF). A value greater than 5 (VIF > 5) would indicate a collinearity problem [69]. The outer VIF values (S1 Table in S1 File) and inner VIF values (S2 Table in S1 File) given in the supporting information are below 5, thus there is no collinearity problem.

To test the hypotheses and determine the association between the psyschological traits and both financial behavior and financial well-being of the young adults, path coefficients were measured. Path coefficients represent the direct effect of a variable on another variable. A value close to 1 shows a stronger association while the reverse is true for a value closer to 0 i.e. a weak relationship. Values close to zero are not statistically significant [69]. Table 4 shows the association between the dependent and independent variables.

**Table 4:** Path coefficients of model.

Relationship	Hypotheses	Beta values	Mean	STDEV	t values	P Values	Decision
Self-Esteem -> Financial Behavior	H_A1_	0.100	0.099	0.064	1.569	0.117	Rejected
Self-Esteem -> Financial Security	H_A5_	0.257	0.260	0.057	4.490	0.000	Accepted
Self-Esteem -> Financial Anxiety	H_A9_	-0.263	-0.270	0.061	4.311	0.000	Accepted
Optimism -> Financial Behavior	H_A2_	0.084	0.085	0.058	1.442	0.150	Rejected
Optimism -> Financial Security	H_A6_	0.076	0.075	0.050	1.495	0.135	Rejected
Optimism -> Financial Anxiety	H_A10_	0.067	0.070	0.059	1.136	0.256	Rejected
Deliberative Thinking -> Financial Behavior	H_A3_	0.164	0.176	0.055	2.956	0.003	Accepted
Deliberative Thinking -> Financial Security	H_A7_	0.130	0.132	0.050	2.584	0.010	Accepted
Deliberative Thinking -> Financial Anxiety	H_A11_	0.037	0.042	0.063	0.577	0.564	Rejected
Self-Control -> Financial Behavior	H_A4_	0.147	0.156	0.056	2.642	0.009	Accepted
Self-Control -> Financial Security	H_A8_	0.148	0.155	0.047	3.152	0.002	Accepted
Self-Control -> Financial Anxiety	H_A12_	-0.054	-0.057	0.061	0.893	0.372	Rejected

To determine if the association is significant, bootstrapping was run to get the significance values where the commonly used critical value for two-tailed tests is p value = 0.05 at significance level of 5%. Based on the p-values, the respective hypotheses were accepted or rejected. Path coefficient values given in Table 4 show that deliberative thinking exhibits a positive association with financial behavior (β = 0.164, p < 0.05) and with financial security (β = 0.130, p < 0.05) while it shows no significant association with financial anxiety (β = 0.037, p > 0.05). Optimism shows no association with financial behavior (β = 0.084, p > 0.05), financial security (β = 0.076, p > 0.05) and financial anxiety (β = 0.067, p > 0.05). Self-esteem shows no association with financial behavior (β = 0.100, p > 0.05) while it exhibits a positive association with financial security (β = 0.257, p < 0.05) and a negative association with financial anxiety (β = -0.263, p < 0.05). Finally, Self-control shows a positive significant association with financial behavior (β = 0.147, p < 0.05) and with financial Security (β = 0.148, p < 0.05) while it exhibits a non-significant negative association with financial anxiety (β = -0.054, p > 0.05).

Table 5 shows the values of R^2^ for financial behavior, financial security and financial anxiety. R^2^ tells about the variability in a dependent variable due to the independent variables. The value of R^2^ ranges from 0 to 1, the value being closest to 1 predicting more accuracy than the value close to 0. According to Chin [70]:

- R^2^ = 0.67 is strong
- R^2^ = 0.33 is moderate
- R^2^ = 0.19 is weak

**Table 5:** Coefficient of determination (R2) of model.

	R Square	P Values
Financial Behavior	0.121	0.000
Financial Security	0.198	0.000
Financial Anxiety	0.063	0.010

The coefficients of determination, R^2^ = 0.121, R^2^ = 0.198 and R^2^ = 0.063 represent a variability of 12.1%, 19.8% and 6.3% in the financial behavior, financial security and financial anxiety respectively, explained by the independent variables. R^2^ values given in Table 5, represent weak predicting values but this low value does not necessarily indicate a problem, good models can also have a low R^2,^ as it is almost impossible to predict a high value of R^2^ of an outcome variable like human behavior [71].

In addition to measuring the values of R^2^, it is also important to examine the Stone-Geisser’s Q^2^. The Q^2^ indicates the predictive capability of the model i.e. how accurately the model predicts the dependent variable. The value of Q^2^ is obtained by using Blindfolding technique. A value greater than 1 indicates that the model has predictive power for a certain variable while a value 0 or below 0 indicates no predictive power [69]. Table 6 shows the predictive relevance (Q^2^) of the model. Since the Q^2^ values (0.040, 0.106, 0.027) are greater than 0, it indicates that the model has predictive relevance for financial behavior, financial security and financial anxiety respectively.

**Table 6:** Blindfolding and predictive relevance (Q^2^) of model.

	SSO	SSE	Q² (= 1-SSE/SSO)
Deliberative Thinking	6435.000	6435.000	
Financial Anxiety	2145.000	2087.724	0.027
Financial Behavior	3432.000	3293.259	0.040
Financial Security	2145.000	1917.729	0.106
Optimism	1287.000	1287.000	
Self-Control	3861.000	3861.000	
Self-Esteem	3861.000	3861.000	

## 4. Discussion and conclusion

This research seeks to determine how the financial behavior and financial well-being of the young adults are formed, in the light of the identification of the role played by non-cognitive factors. Based upon the literature four such important non-cognitive factors, self-esteem, optimism, deliberative thinking and self-control have been identified. To achieve this goal, a sample of 429 university students was collected via both online and field survey using purposive sampling technique.

SPSS and PLS-SEM were used for the exploration of the relationships among the dependent and independent variables. Demographic profile shows that majority of the respondents were females making up 74.4% of the total sample. Major portion of the respondents belonged to the age groups of 18–22 and 23–26 forming 47.6% and 47.1% of the entire sample population respectively. Majority of the students were from the BS 4-years level (45.5%) while demographics also included the level of monthly household income, where the income of majority of the participants was observed to be between Rs.50,001 and Rs.100,000.

The results of the PLS Model depict a weak association among the dependent and independent variables as can be seen from the values of coefficient of determination (R^2^ = 0.121, R^2^ = 0.198 and R^2^ = 0.063), exhibiting a variability of 12.1%, 19.8% and 6.3% in the financial behavior, financial security and financial anxiety respectively, due to the independent variables (non-cognitive factors). PLS path analysis illustrates that self-control and deliberative thinking show a significant positive association with both financial behavior and financial security and no association with financial anxiety, this is in line with the previous studies [3, 13, 48]. Self-esteem shows no relationship with financial behavior when all the variables are taken together but it exhibits a positive association with financial security and a negative association with financial anxiety, showing that financial anxiety decreases with an increase in the self-esteem. Optimism on the other hand exhibits no association with financial behavior, financial security and financial anxiety. The insignificant association between optimism and financial behavior is in line with previous studies [13, 48].

When all the predictor variables are taken together in PLS-SEM to know their combined effect on financial behavior and financial well-being, the effect of self-esteem and optimism reduces to the extent that the relationship with financial behavior and well-being (in case of optimism) becomes insignificant. The results of PLS-SEM path analysis shows that self-control, deliberative thinking and optimism play no significant role in reducing the financial anxiety of the individuals. While on the other hand individuals exhibiting self-control and deliberateness observe better financial behavior and feel more secured about their financial situations.

The differences in the results from the previous studies can be explained based on cultural variations as moving from one region to another, cultural differences become dominant. People living in Pakistan belong to a consumption-oriented economy where no strict future planning in terms of financial security is made and thus, people save less. Retirement planning is rare, and insurance is still an unfamiliar concept to the common man. Therefore, a weak relation among the dependent and independent variables may be because of these few reasons. As far as the financial anxiety is concerned, along with the above reasons stated, one reason for causing high anxiety levels and less optimistic approach of the people might be that, since the data was collected during the period of COVID-19 wherein the individuals around the world were suffering from various psychological disorders including, increased levels of anxiety and clinical depression along with global economic downturn; might have influenced the responses of the individuals.

## 5. Limitations and recommendations

Some of the limitations of the present study are: data collection done via online survey exhibit a self-report inventory. One of the problems with self-reported data is that there is a possibility for the respondents to misunderstand the questions or intentionally or unintentionally give wrong answers according to what they think is desired for a particular question thus, causing social desirability bias. Secondly, since the data was collected during the time of a global pandemic, COVID-19, which might have affected individual’s both physically and mentally thus, in turn influencing their responses.

The instrument used for measuring the general financial behavior consisted of some question related to loans payments, credit cards and their usage, such behaviors are relatively rare for the students (with some exceptions), though these items were provided with an option of “Not Applicable”. Future studies should make use of such questions that better describe the financial behavior of students. Moreover, the sample only consisted of university students, future studies should make use of a sample population other than the students such as entrepreneurs, who manage small or medium level businesses and play a role in the economy of the country. There are a large number of other non-cognitive factors such as intuition, locus of control and self-efficacy, future studies should also throw light on the roles played by these factors on the financial capabilities and well-being of the individuals. After the pandemic is over, this study could be conducted to learn about the financial behavior and financial well-being of the individuals which they would exhibit under normal circumstances.

## 6. Implication

This study contributes to the economic enrichment especially as it highlights the importance of financial behavior and financial well-being of the young adults by including non-cognitive factors. Such a study, exploring the role of non-cognitive factors like self-esteem, optimism, deliberative thinking and self-control, has not been addressed considerably in Pakistan. This research study makes a number of contributions to the existing literature i.e. (i) this study is closely related to the literature which explores the determinants of financial behavior and financial well-being, especially in case of self-esteem as its association with financial behavior and financial well-being in the presence of self-control, optimism and deliberative thinking has not been addressed in particular (ii) most of the former research focused on cognitive factors such as financial literacy, but this study focuses on non-cognitive factors. Through this study we show that these factors are equally important as the cognitive factors in determining the financial behavior and financial well-being of the individuals.

Our findings have important implications for the education sector, policy makers and the government to help them in their struggle of improving financial management. The financial education programs centered on instilling financial literacy to achieve the goal of improving financial behavior of the individuals should also emphasize focus on the development of non-cognitive or psychological factors [9], for the better development of financial behavior and well-being. As the students are the future market bearers and assets of a country, more emphasis should be given to the development of these soft skills along with financial literacy of the students to enable them make better financial decisions, leading to better a well-being.

## Supporting information

S1 File (ZIP)
