# Supplementary material for: The role of self-esteem, optimism, deliberative thinking and self-control in shaping the financial behavior and financial well-being of young adults
Source: PLoS One. 2021 Sep 7;16(9):e0256649. doi: 10.1371/journal.pone.0256649 (PMC8423263; doi:10.1371/journal.pone.0256649)
Supplement: S1 File — (ZIP) [file pone.0256649.s001.zip › Supporting information/S1_Table.pdf]

S1 Table: Collinearity Assessment: Outer VIF Values

Constructs	Item Codes	Outer VIF Values
Self-Esteem		
	SE1	1.419
	SE2	1.459
	SE3	1.606
	SE4	1.492
	SE5	1.438
	SE6	1.344
	SE7	1.439
	SE9	1.523
	SE2	1.459
	SE10	1.482
Optimism		
	O1	1.481
	O4	1.294
	O10	1.322
Deliberative Thinking		
	DT1	1.578
	DT2	1.522
	DT3	1.640
	DT4	1.584
	DT5	1.544
	DT6	1.714
	DT7	1.868
	DT8	1.848
	DT9	1.745
	DT10	1.908
	DT11	1.749
	DT12	1.502
	DT13	2.113
	DT14	2.281
	DT15	1.575
Self-Control		
	SC2	1.227
	SC3	1.396
	SC4	1.417
	SC5	1.535
	SC7	1.299

	SC9	1.520
	SC10	1.526
	SC12	1.508
	SC13	1.330
Financial Behavior		
	FB2	1.228
	FB3	1.183
	FB4	1.316
	FB5	1.298
	FB8	1.478
	FB9	1.829
	FB10	1.874
	FB13	1.310
Financial Security		
	FWB1	1.957
	FWB2	2.461
	FWB3	1.707
	FWB4	1.642
	FWB5	1.614
Financial Anxiety		
	FWB6	1.576
	FWB7	1.709
	FWB8	1.754
	FWB9	1.973
	FWB10	1.569
