# Supplementary material for: The role of self-esteem, optimism, deliberative thinking and self-control in shaping the financial behavior and financial well-being of young adults
Source: PLoS One. 2021 Sep 7;16(9):e0256649. doi: 10.1371/journal.pone.0256649 (PMC8423263; doi:10.1371/journal.pone.0256649)
Supplement: S1 File — (ZIP) [file pone.0256649.s001.zip › Supporting information/S2_Table.pdf]

S2 Table: Collinearity Assessment: Inner VIF Values

	Financial Behavior	Financial Security	Financial Anxiety
Deliberative Thinking	1.234	1.234	1.234
Optimism	1.356	1.356	1.356
Self-Control	1.174	1.174	1.174
Self-Esteem	1.440	1.440	1.440
